# Trends over Time in Dental Caries status in Urban and Rural Thai Children 

**DOI:** 10.4317/jced.54054

**Published:** 2017-10-01

**Authors:** Patcharawan Srisilapanan, Areerat Nirunsittirat, Jeffrey Roseman

**Affiliations:** 1 Center of Excellence in Dental Public Health, Faculty of Dentistry, Chiang Mai University, Chiang Mai, Thailand; 2 Professor Emeritus, Department of Epidemiology, UAB School of Public Health, University of Alabama at Birmingham, Birmingham, Alabama, USA

## Abstract

**Background:**

Historically, the prevalence of dental caries was higher in urban areas than rural areas of Thailand. This study aim to examine the time trends in caries status in children in Thailand.

**Material and Methods:**

Linear regression was used to examine trend of dental caries prevalence and mean number of teeth with caries, filled and missing due to caries (dmft/DMFT) in urban and rural, of 3-, 5-6 and 12-year olds from seven Thailand National Oral Health Surveys conducted approximately every 5 years from 1977 to 2012.

**Results:**

There were declines in the caries prevalence and mean dmft/DMFT in every age group. Significant results were observed in the mean dmft of 3 year-olds and the mean DMFT of 12 year-olds (*p*= 0.03 and *p*=0.05, respectively). A significant trend of declining prevalence of dental caries was observed in urban children ages 5-6 (*p*=0.002), along with urban 12 year-olds (*p*<0.001). A declining trend of mean dmft for 3 and 5-6 year-olds, and mean DMFT for 12 year-olds was observed in both rural and urban areas, but significant results were shown in urban 3 and 5-6 year-olds (*P*=0.04, and *p*<0.001, respectively), and urban 12 year-olds (*p*=0.001). For restoration outcome, both urban and rural of all age groups have an increasing trend of mean ft/FT index.

**Conclusions:**

There have been differences over time in the prevalence and quantity of dental caries between urban and rural school children. A significant reduction was observed in urban areas. More effort needs to be given to supply rural areas in order to have fair and equal access of all citizens to oral health care.

** Key words:**Dental caries, prevalence, children, Thailand, rural, urban, time trend, national survey.

## Introduction

The Thailand National Oral Health Survey (TNOHS) provides an estimate of oral health status; an assessment of the burden of selected oral diseases; and an explanation of determinants of oral health and known risk factors. The first TNHOS was conducted in 1977 by the Ministry of Public Health under the responsibility of the Dental Health Bureau. Since the second survey in 1984, it has been conducted every five years. The most recent TNHOS was in 2012 (1). During the past several decades, the Bureau of Dental Health, under the Ministry of Public Health, has implemented several oral health projects based the information from the TNHOS for pre-school children, school children, and other age groups. In the earliest studies of caries prevalence of children in Thailand, their prevalence was higher in urban than in rural areas (2-4). Similarly the data from the first TNOHS in 1977 reported the prevalence of dental caries in 3, 5-6, and 12 year-old children was higher in urban areas than rural areas (1).

Since the first TNOHS, there has been a marked increase in sugar intake (5), in both urban and rural Thailand. This may have an effect on the prevalence of dental caries in Thai children. However, the trend of dental caries had not been explored. The purpose to this study was to determine the trends over time in the prevalence of dental caries and assess the difference in the trends of dental caries between children of urban and rural areas.

## Material and Methods

The data used in these analyses comes from seven TNOHSs (1,6-9). The sample includes three age groups, 3, 5-6, and 12 year-olds. There were 23,319 3 year-olds, 34,412 5 year-olds, and 47,111 12 year-olds are included in the analysis.

The first national survey was started in 1977. The 2nd to 7th surveys were conducted every five years from 1984 to 2012. Stratified multi stage sampling was used. The first stage was to stratify the country into five regions: Northern, Northeastern, Central, Southern, and Bangkok Metropolitan. Systematic sampling was used to sample four provinces from each region. There were 17 provinces including Bangkok Metropolitan in the survey. In each province, stratified sampling according to the definition of the Ministry of Interior was 1:2 for urban and rural areas, 1:1 for each province, and 1:1 male number to female (1).

Since the 3rd TNOHS, the methods used for dental examination, diagnosis, and reporting of caries prevalence have followed the guidelines of the World Health Organization’s (WHO) 3rd, 4th and 5th edition of the Basic Methods for Oral Epidemiology Studies (10-12). Each tooth was determined and recorded as sound, decayed, missing due to decay, or filled due to decayed. Caries experiences were calculated by determining the number of decayed, missing, or filled teeth (dmft for primary teeth and DMFT for permanent teeth). Although the conductors of the measurements have changed, the training program has remained consistent. Since the 5th National Oral Health Survey, eight examining teams have been appointed to conduct the survey. The same team has been responsible for the 6th and 7th survey (1).

The data were analyzed by SPSS version 22.0 (18). Linear regression analyses were used to assess caries prevalence trends and to describe mean dmft/DMFT, and f/t or F/T by year of survey, and urban/rural areas. The significance threshold was set as *p*<0.05.

## Results

A total of 104,847 of 3, 5-6 and 12 year-old children were included in the analyses. The reported results of dental caries prevalence, and mean caries experience (mean dmft and DMFT) by age, urban/rural status, number of children included in each survey, and the year of the national survey and are presented in  Table 1. There were declines in the dental caries prevalence and mean dmft/DMFT of every age group, however significant results were observed only in the mean dmft of 3 year-olds and the mean DMFT of 12 year-olds (*p*= 0.03 and *p*=0.05 respectively).

**Table 1:**
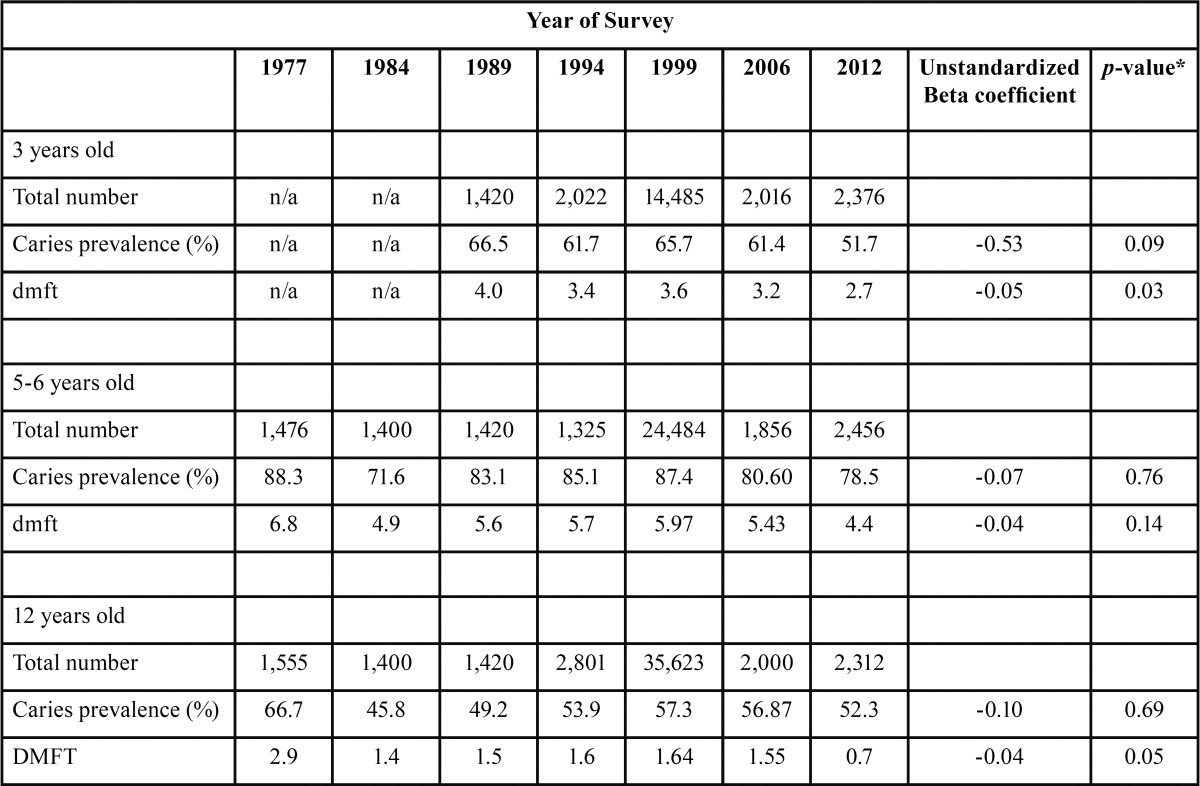
Total number of children examined, dental caries prevalence and mean dmft/DMFT by age group and year of study.

**Figure 1:**
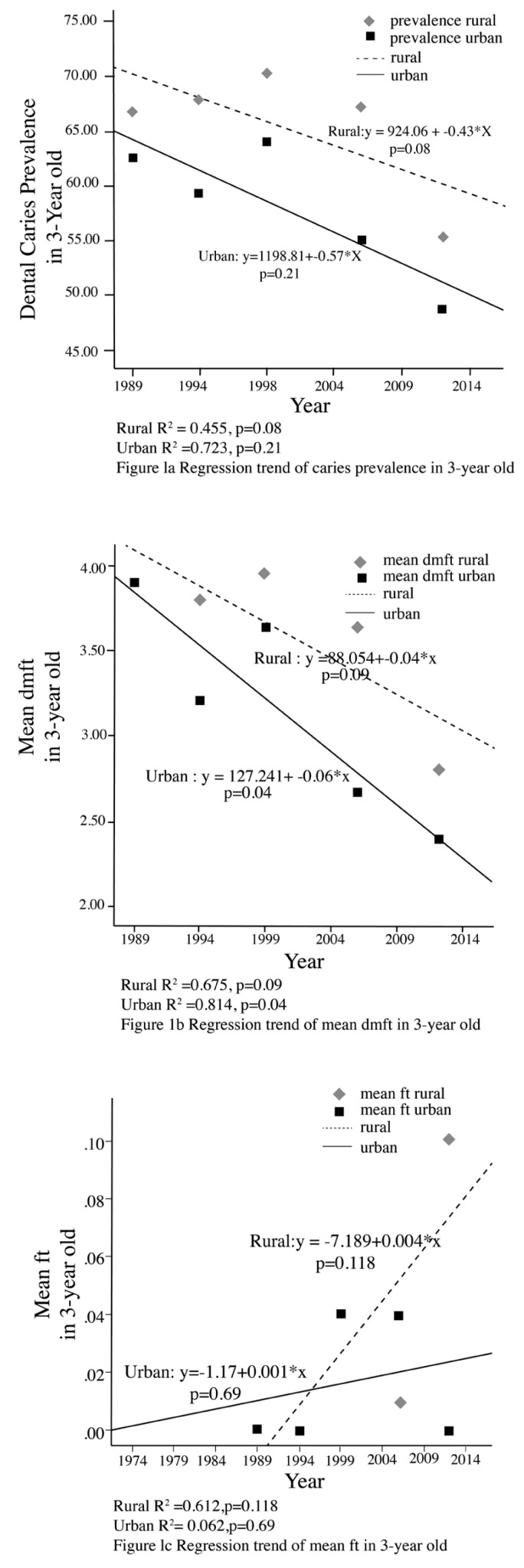
Regression trend of caries prevalence and mean dmft in 3-year old in Rural vs Urban.

**Figure 2:**
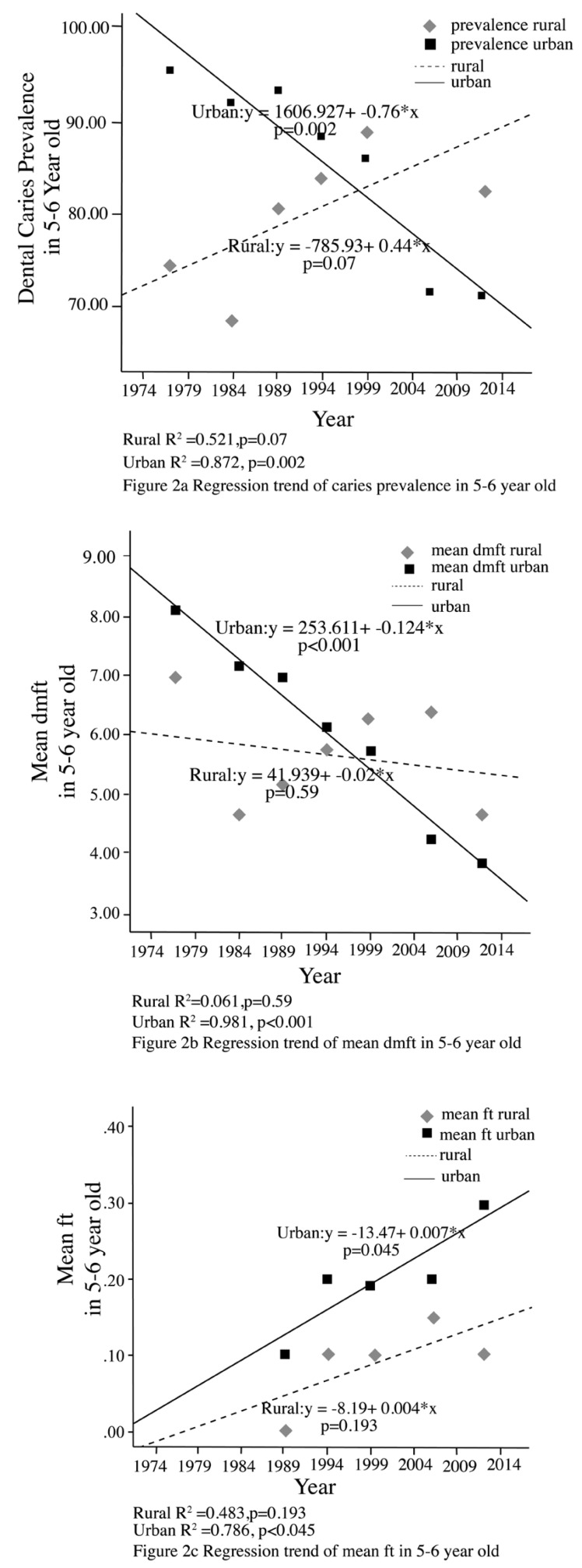
Regression trend of caries prevalence and mean dmft in 5-6 year olds in Rural vs Urban.

**Figure 3:**
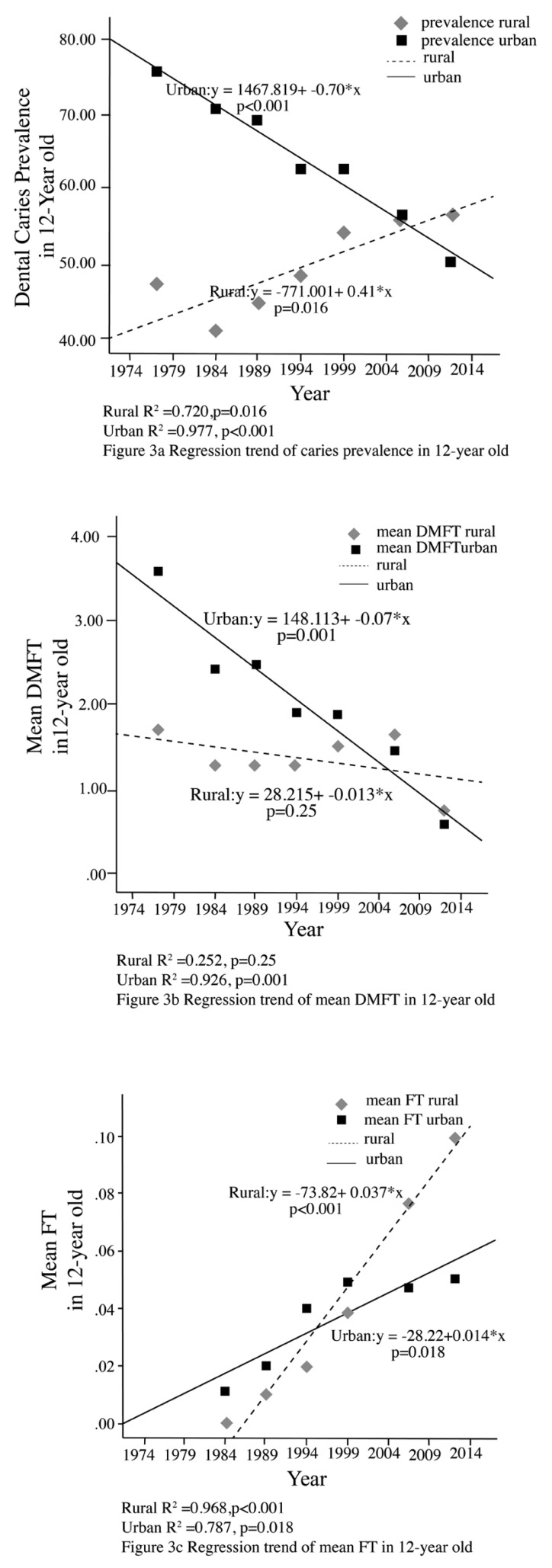
Regression trend of caries prevalence and mean DMFT in 12-year olds in Rural vs Urban.

A comparison of the reported results of caries prevalence and mean dmft/DMFT in rural and urban areas is shown in Figures 1-3. Among 3 year-olds there have been non-significant declines in the prevalence of dental caries in both rural and urban areas (Fig. 1). With respect to 5-6 year olds, there have been significant declines in the prevalence of dental caries among those from the urban areas (p=0.002), and a non-significant increase among those in rural areas (*p*=0.07) although the most recent survey showed a decline (Fig. 2). Among 12-year olds there was found to be significant declines in dental caries prevalence in those from urban areas (*p*<0.001) and a significant increase in those from rural areas (*p*=0.016) (Fig. 3).

A comparison of the mean dmft/DMFT of rural and urban areas is shown in Figures 1-3. Among 3 year-olds there has been a non-significant decline in mean dmft in rural areas (*p*=0.09). A significant decline in the mean dmft was found in the urban sample (*p*=0.04) (Fig. 1). With respect to 5-6 year-olds, there has been a significant decline in mean dmft only among those from the urban areas (*p*<0.001). A non-significant decline was found in the mean dmft in rural areas (*p*=0.59) with the most recent survey showing a sharp decline (Fig. 2). With respect to 12-year olds there has been a significant decline in the mean DMFT (*p*=0.001) among those from urban areas while in rural areas mean DMFT decreased, but with non-significant results (*p*=0.25) (Fig. 3).

For restoration outcome, a comparison of the mean ft/FT of rural and urban areas is shown in Figures 1, 2 and 3. There has been increase in the mean filled teeth in both urban and rural area of all age groups. A significant increase in the mean ft/FT were found in urban 5-6 year-olds (*p*=0.045) (Fig. 2), rural and urban 12 year-olds (*p*=0.018 and *p*<0.001 respectively) (Fig. 3).

## Discussion

There are many possible explanations for these findings. While further research is necessary to determine the forces responsible for these trends, we can speculate as to the explanation(s). It is certainly possible that the trends reflect sampling or measurement differences over time. However, given the consistency of the results across age groups it seems unlikely that sampling differences are responsible given the opposite directions of the regressions. It thus seems unlikely that the results are due to measurement differences. Our results are also consistent with previous studies, not related to the TNOHS, which showed trends of increasing prevalence in rural areas in 5-6 and 12 year-olds. In the earliest study of caries prevalence in children in Thailand, conducted in 1951 by Kridakara *et al.*, the prevalence in the Bangkok urban area was considerably higher than in Chiang Mai and Lamphun (considered rural areas at that time) (2). A survey conducted in urban and rural Thailand in 1986 found the prevalence of dental caries was higher in Bangkok than in the rural areas (3). Similar urban/rural differences were found in a study conducted in Southern Thailand in 1997 (4).

In our analysis, we found it remarkable that dental caries prevalence, and mean dmft/DMFT declined for all age groups in urban areas. Despite public efforts to reduce the consumption of sugar (13), there has been a marked increase in sugar intake (5) over the period of time reviewed in the survey. Furthermore, the TNONS found that the amount of candy eaten daily is very similar between urban and rural 12-year olds. This is similar to trends reported in many industrialized nations, where the prevalence of dental caries has declined despite an increase in sugar intake (14,15). This would suggest that sugar intake cannot be the sole factor in determining dental caries prevalence. One possible explanation might be exposure to fluoride containing dentifrices. However, this would not account for the urban decline in 3-year olds since they are too young to be using fluoride-containing toothpaste. Another possible explanation is increased good oral health habits in the urban areas (16), although there is scant evidence for this.

The national survey shows an increased percentage of daily brushing before bed in urban areas, however the data is only for 12-year olds and there is no evidence of a trend (results not shown). Also, urban areas had a higher percentage of daily brushing before bed at a time when caries prevalence was higher in the urban areas.

Another possible explanation is the number of dentists in relation to the population. In 1990, 2000, 2005, 2013 and 2015, the dentist per population ratio were 1:21609, 1:9800, 1:7380, 1:5359 and 1:4913 respectively (17,18). Although the density of dentists was lower in rural regions than in urban regions (17) from 1990 to 2015, the ratio of dentists to people has increased from 1:21,609 to 1:4,913 (18). The increase of dentists per capita occurred in both rural and urban areas, so this is not consistent with the increase in caries only in rural children. The availability of dental services does not seem to be an explanation. Studies in other countries have shown that dental services have a minimal effect (14,15) on caries prevalence. Increasing the number of dentists does not solve the dental health problem. What is needed is even distribution of medical care in urban and rural areas. Currently, it is much more lucrative for dentists to open practices and work in urban areas. Until the distribution of dentists across the country is equal, inequalities in dental health will persist.

Another possible contributing factor to the decline in caries prevalence is the increased number of dental nurses working in rural areas. Dental nurse in Thailand could be able to provide simple dental treatment such as filling, extraction and scaling for children equal or below 12 years old (19). This may be the attributing factor for the increased number of fillings in the dft/DFT trends). However, further research needed to investigate these impacts.

If improvement in oral health among urban children is attributable to improvements in oral hygiene, it is important to know if it reflects changes in parental behavior and if so, if government, health care professionals and/or academics have had influences. Further studies are necessary to determine explanations for these trends.

There is an interesting trend to observe in the comparison of rural caries prevalence and dmft/DMFT. In 5-6 and 12 years-old rural populations, while the prevalence of caries shows an increasing trend, the mean dmft/DMFT and ft/FT indices show a decreasing trend. One possible explanation is the increased access to dental services available in the rural areas. Other studies have shown (20) that such trends can be found when people have increased access to dental services. Further study is needed to find an explanation for this anomaly in the data.

It is certainly possible that the increase in sugar intake and the absence of good oral hygiene in rural areas is responsible for the overall increase. However, recent decreases in caries prevalence in rural and 3 year-olds give reason for optimism. One possible explanation is public exposure to milk with fluoride. In 2000, the World Health Organization, along with the Borrow Milk Foundation, implemented a milk fluoridation project in eight provinces which covered approximately one million school children. One study found a 34.4 percent reduction rate of dental caries in school children who consumed fluoridated milk compared to those who consumed non-fluoridated milk after five years (21). Widely use of fluoride toothpaste in Thailand could be the major reason for decrease dental caries prevalence. It will be important to see if this trend continues.

## Conclusions

There have been differences over time in the prevalence and quantity of dental caries between urban and rural school children, however very recent declines in rural children give reason for optimism. It is important to try to identify the salient factors leading to the observed trends in order to reduce caries prevalence. In Thailand, there is disproportionate access to dental services between rural and urban populations. More effort needs to be given to supply rural areas with dental care in order to have fair and equal access of all citizens to medical services.
